# Beneficial effects of rapamycin on endothelial function in systemic lupus erythematosus

**DOI:** 10.3389/fphys.2024.1446836

**Published:** 2024-08-21

**Authors:** Hyoseon Kim, Michael P. Massett

**Affiliations:** Department of Kinesiology and Sport Management, Texas Tech University, Lubbock, TX, United States

**Keywords:** inflammatory response, lpr mice, vascular function, mouse aorta, mTORC1 inhibition, mitophagy

## Abstract

**Introduction:**

Endothelial function is significantly impaired in patients with SLE compared to healthy controls. Elevated activation of the mammalian target of rapamycin complex 1 (mTORC1) is reported in humans and mice with SLE. However, it is unclear if elevated mTORC1 in SLE contributes to impaired mitophagy and endothelial dysfunction. Therefore, we tested the hypothesis that inhibiting mTORC1 with rapamycin would increase mitophagy and attenuate endothelial dysfunction and inflammatory responses in SLE.

**Methods:**

Nine-week-old female lupus-prone (MRL/lpr) and healthy control (MRL/MpJ) mice were randomly assigned into rapamycin treatment (lpr_Rapamycin and MpJ_Rapamycin) or control (lpr_Control and MpJ_Control) groups. Rapamycin was injected i.p. 3 days per week for 8 weeks. After 8 weeks, endothelium-dependent vasorelaxation to acetylcholine (ACh) and endothelium-independent vasorelaxation to sodium nitroprusside (SNP) were measured in thoracic aortas using a wire myograph.

**Results:**

MTORC1 activity was increased in aorta from lpr mice as demonstrated by increased phosphorylation of s6rp and p70s6k and significantly inhibited by rapamycin (s6rp, *p* < 0.0001, p70s6k, *p* = 0.04, respectively). Maximal responses to Ach were significantly impaired in lpr_Control (51.7% ± 6.6%) compared to MpJ_Control (86.7% ± 3.6%) (*p* < 0.0001). Rapamycin prevented endothelial dysfunction in the thoracic aorta from lupus mice (lpr_Rapamycin) (79.6% ± 4.2%) compared to lpr_Control (*p* = 0.002). Maximal responses to SNP were not different across groups. Phosphorylation of endothelial nitric oxide synthase also was 42% lower in lpr_Control than MpJ_Control and 46% higher in lpr_Rapamycin than lpr_Control. The inflammatory marker, vascular cell adhesion protein 1 (Vcam 1), was elevated in aorta from lupus mice compared with healthy mice (*p* = 0.001), and significantly reduced with Rapamycin treatment (*p* = 0.0021). Mitophagy markers were higher in lupus mice and reduced by rapamycin treatment, suggesting altered mitophagy in lpr mice.

**Conclusion:**

Collectively, these results demonstrate the beneficial effects of inhibiting mTORC1 on endothelial function in SLE mice and suggest inflammation and altered mitophagy contribute to endothelial dysfunction in SLE.

## 1 Introduction

Emerging evidence suggests dysfunctional mitochondria contribute to immune dysregulation and multiple organ damage such as kidney, liver, lung, skin, and blood vessels in systemic lupus erythematosus (SLE) (Perl et al., 2012). Besides organ damage, SLE is characterized by having an increased cardiovascular disease (CVD) risk due to both traditional CVD risk factors and non-traditional, SLE-related factors. Treatment options for SLE patients include steroids, immunosuppressive drugs, and disease-modifying anti-rheumatic drugs, which are reported to target the inflammatory responses in SLE (Trager and Ward, 2001; Davis and Reimold, 2017). Although deaths caused by SLE activity decline with long-term treatment using immunosuppressive drugs, a 7- to 10- fold higher risk of cardiovascular disease (CVD) has been reported in SLE patients (Manzi et al., 1997). Furthermore, some immunosuppressive drugs are reported to have detrimental effects on endothelial function and vascular damage in SLE (Tepperman et al., 2010). Regardless of treatment, endothelial dysfunction is commonly reported in SLE patients (Mauro and Nerviani, 2018). Endothelial dysfunction is theoretically reversible. Thus, there is a significant necessity to investigate therapeutic approaches of preventing endothelial dysfunction and attenuating the risk of CVD in SLE.

The mammalian target of rapamycin (mTOR) regulates mitochondrial quality control by stimulating mitochondrial biogenesis, fission, and fusion, and inhibiting mitophagy (Shimobayashi and Hall, 2014). According to some studies that used genetically modified mice, mTORC1 activity is also reported to be a critical regulator of endothelial function (Reho et al., 2019; Islam et al., 2024). Elevated mTORC1 activity has been shown in SLE patients and mice (Gergely et al., 2002; Perl, 2010), suggesting an inhibition of mTORC1 as a potential therapeutic target to prevent impaired endothelial function of SLE. However, elevated levels of mTOR were not directly related to endothelial dysfunction in obesity (Reho et al., 2021), which points to a discrepancy between pathologically and experimentally induced mTORC1 activity on endothelial function. It is still unclear if pathologically elevated mTORC1 in SLE results in endothelial dysfunction. Thus, the purpose of this study was to determine the effect of inhibition of mTORC1 on endothelial dysfunction in SLE.

Elevated mTOR complex 1 (mTORC1) impairs mitochondrial function by inhibiting mitophagy (Bartolome et al., 2017). Impaired mitophagy is associated with defective clearance of damaged mitochondria releasing Damaged Associated Molecular Patterns (DAMPs). DAMPs are molecules released into the extracellular space under the circumstances of dying and dead cells. DAMPs include oxidized mitochondrial DNA (Ox-mtDNA), cardiolipin, and mitochondrial transcription factor A (TFAM). DAMPs amplify inflammatory responses by binding to the Pattern Recognition Receptors (PRRs). SLE is associated with markedly increased levels of DAMPs. Such viral pathogens cause signaling through Toll-like Receptors (TLRs) which leads to the production of interferons and inflammatory cytokines, resulting in chronic inflammation disease. The oxidized mtDNA, one example of DAMPs, was reported to produce autoantibodies and type 1 interferon (Type I IFN) in SLE patients (Xu et al., 2020). One of the DAMPs that can be recognized as an autoantigen in SLE is cell-free DNA (cfDNA) (Pisetsky, 2012). The antibodies to double-stranded DNA are considered diagnostic for SLE (de Leeuw et al., 2017). Therefore, targeting the clearance of damaged cells could be a potential therapeutic strategy to reverse lupus-associated inflammation and organ damage (Palikaras et al., 2017).

Rapamycin, which enhances the removal of damaged mitochondria, reportedly attenuates mTOR (mammalian target of rapamycin) activity, preserving energy metabolism through clearing damaged mitochondria and activation of mitochondrial biogenesis. Increasing results support the efficacy of rapamycin for alleviating lupus disease activities, protecting organ damage, and extending life span both in humans and rodents (Fernandez et al., 2006; Yap et al., 2012; Lai et al., 2013; Liu et al., 2015; Oaks et al., 2016). Inhibiting mTORC1 has been shown to reduce inflammatory responses, preventing further damage in the kidney, liver, and spleen in clinical studies. For example, 12-month treatment with the rapamycin analog sirolimus in lupus patients resistant to standard treatments improved multiple markers of disease activity (Lai et al., 2018). Research in animals suggests that rapamycin can inhibit the production of autoantibodies, improve proteinuria, and block the progression of lupus nephritis (Ramos-Barrón et al., 2007; Gu et al., 2016). On the other hand, the effect of rapamycin on vascular function has been controversial. Some studies showed the beneficial effect of rapamycin on improving vascular function and reducing atherosclerosis in *ex vivo* aortic rings with mTORC1 activation, and also in animal models with diabetes and eNOS-eGFP transgenic mice (Cheng et al., 2008; Das et al., 2014; Reho et al., 2019). Other studies, however, have demonstrated a negative effect of inhibiting mitophagy on endothelial function. Patients with hyperlipidemia treated with rapamycin experienced impaired endothelial function by reduced NO production and cell migration (Fruhwurth et al., 2014). Kurdi et al. (2016) suggested that rapamycin reduced insulin sensitivity and increased peripheral insulin resistance which can negatively affect endothelial function. None of these studies have utilized rapamycin treatment in SLE populations or lupus-prone mouse models to evaluate its effect on endothelial dysfunction. Based on rapamycin’s potential benefit on lupus disease activity in general, this study was conducted to determine the effect of rapamycin on mitophagy and endothelial function in this specific disease.

## 2 Materials and methods

### 2.1 Ethics approval

Before starting the study, the study was approved by the Texas Tech University Institutional Animal Care and Use Committee. All procedures were performed under the Public Health Service’s Policy on Human Care and Use of Laboratory Animals guidelines.

### 2.2 Animal

Female mice from strains MRL/lpr and MRL/MpJ (n = 30/strain) were purchased from Jackson Laboratories (Bar Harbor, ME) and housed at the animal facility at Texas Tech University. Only female mice were used because the disease is more prevalent in female mice compared with males (Gao et al., 2010). All mice were received at 6 weeks of age and allowed to acclimate for 1–2 weeks prior to use. Mice were housed in the same room under standard conditions (non-barrier), maintained on a 12:12 h light-dark cycle in a controlled temperature (21°C–22°C), and allowed food (Standardize Laboratory Rodent Diet) and water ad libitum. At 9 weeks old, mice were randomly separated into two groups- treatment or control. The treatment group was given rapamycin by intraperitoneal injection three times a week for 8 weeks. The control group was injected with the vehicle. At 16 weeks of age, mice were weighed and anesthetized by intraperitoneal injection of a cocktail of ketamine (80 mg/kg) and xylazine (5 mg/kg). The serum samples were collected by cardiac puncture. The thoracic aortas were harvested for functional analysis. The abdominal aorta, heart, kidney, liver, and spleen were harvested for gene expression and Western blot analysis. Tissues were weighed before being stored. Spleen were removed and their weight and length were measured. The whole tissues were snap-frozen in liquid nitrogen and stored at −80°C until utilized for the analysis.

### 2.3 Urinary protein

Urine samples were collected from each mouse every week after the initiation of drug treatment to assess proteinuria. Urinary protein excretion was analyzed semi-quantitively as grade 0 (negative), grade 1 + (≥30 mg/dL), grade 2 + (≥100 mg/dL), grade 3 + (≥300 mg/dL), and grade 4 + (≥2000 mg/dL) using a colorimetric assay (Albustix, Bayer) according to the manufacturer’s recommendations. Urine samples with 300 mg/dL or more were defined as proteinuria.

### 2.4 Intraperitoneal injection of rapamycin

The treatment group was treated with 1 mg/kg rapamycin (LC Laboratories, #R-5000). Rapamycin (n = 15/strain) or vehicle (n = 15/strain) was injected intraperitoneally (∼100 uL/10 g) 3 times a week. Rapamycin solution was prepared by dissolving rapamycin stock solution (prepared in DMSO) in 5% PEG-400/5% TWEEN 80 (vehicle) to a concentration of ∼1.2 mg/mL (Bitto et al., 2016). Control mice were injected with a vehicle containing an equal volume of diluent DMSO without rapamycin.

### 2.5 Vasoreactivity assessment

#### 2.5.1 Isolation of arteries

Thoracic aortas (TA) were isolated. Connective tissue and perivascular adipose tissue were carefully removed in ice-cold physiological saline solution pH 7.4 (in mM: 118.31 NaCl, 4.69 KCl, 1.2 MgSO_4_, 1.18 KH_2_PO_4_, 24.04 NaHCO_3_, 0.02 EDTA, 2.5 CaCl_2_, and 5.5 glucose) under a microscope. Vessels were cut into 2 mm ring segments of equal length. Each ring segment was suspended in an organ chamber of 620 M Multi Chamber Myograph System (Danish Myo Technology, Aarhus, Denmark) filled with 8 mL of oxygenated (95% O_2_, 5% CO_2_) physiological saline solution and allowed to equilibrate at 37°C for at least 30 min.

#### 2.5.2 Functional evaluation

Optimal resting tension was determined based on the standard normalization procedures for wire myography (McPherson, 1992). Aortic ring segments were passively stretched in an incremental manner until the calculated transmural pressure reached 13.3 kPa (100 mmHg). Resting tension was set to an internal circumference of approximately 90% of that at 13.3 kPa. Two aortic rings from each mouse were used. The responses from the vessel segments were averaged before statistical analysis. Aortic ring segments were treated with a single concentration of a selective α1 adrenergic receptor agonist, phenylephrine (PE) (10^−6^ M) to confirm that the aorta was viable. Cumulative concentration-response curves to PE (10^−9^–10^−5^ M in full log increments) and potassium chloride (KCl, 5–100 mM) were generated to assess contractile function. Cumulative concentration-response curves to acetylcholine (ACh, a muscarinic receptor agonist) (10^−9^–10^−5^ M in half-log increments) and sodium nitroprusside (SNP, a nitric oxide donor) (10^−9^–10^−5^ M in full-log increments) were generated to assess endothelium-dependent and independent vasorelaxation, respectively. Concentration-response curves to ACh and SNP were generated after each ring was pre-contracted to 70% of maximum with PE. Percent contraction responses were calculated as [(D_P_ - D_B_)/D_B_] × 100, where D_P_ is the maximal force generated by PE and D_B_ is the baseline force. Percent relaxation responses were calculated as [(D_P_ - D_D_)/(D_P_ - D_B_)] × 100, where D_P_ is the maximal force pre-generated by PE, D_D_ is the lowest force generated at a given dose of ACh or SNP and D_B_ is the baseline force. Area under the curve (AUC) for PE and KCl was determined for each vessel as a marker of potency and efficacy using GraphPad Prism 10.

### 2.6 Western blot

Proteins were isolated from livers and TA. Tissue lysates were harvested in lysis buffer containing: 25 mmol/L Tris-HCl pH 7.5, 150 mmol/L NaCl, 0.1% (v/v) sodium dodecyl sulphate (SDS), 1% Nonidet-P40 (NP-40), a protease inhibitor cocktail (Roche Applied Science, Barcelona, Spain) and a mix of phosphatase inhibitors (1 mmol/L orthovanadate, 20 mmol/L β-glycerophosphate, 10 mmol/L NaF from Sigma-Aldrich). Protein content was determined with BCA protein assay reagent (Pierce, Rockford, IL, United States), using bovine serum albumin (BSA, Sigma-Aldrich., St. Louis, MO, United States) as standard. Lysates (15–25 μg per lane) were separated by 4%–20% mini-protean TGX precast gels and transferred to polyvinylidene difluoride (PVDF) membranes (Bio-Rad Laboratories, Hercules, CA, United States). After protein transfer, the membranes were stained with Ponceau S solution for 15 min to visualize total protein content and verify uniform protein loading. After destaining the membranes, membranes were blocked with 5% non-fat dry milk in tris buffer solution (TBS) for 1 h at room temperature. Then membranes were incubated overnight at 4°C with monoclonal primary antibodies against LC3A/B (#12741), Vcam1 (#39036), SQSTM1/p62 (#5114), p-p70s6k (Thr389 #9234), p70s6k (#34475), p-s6rp (Ser235/236, #2211), s6rp (#2217), p-ULK1 (Ser757, #14202), and ULK1 (#8054) (1/1,000 for each antibody; Cell Signaling, Danvers, MA, United States), p-eNOS (Ser1177, #612392), and eNOS (#610297) (1/1,000 for each antibody; BD Bio Sciences, Becton, NJ, United States). Appropriate HRP-labelled anti-rabbit (1/2,500; Santa Cruz Biotechnology, Inc., Santa Cruz, CA, United States) secondary antibodies were subsequently used for 1 h at room temperature. Proteins were visualized using WesternBright Sirius HRP chemiluminescence (Advansta) on a Bio-Rad ChemiDoc MP imager. Protein band density was quantified using ImageJ software. Membranes were stripped using 5X Western Re-probe for 1 h 30 min and reblotted with antibodies.

### 2.7 Enzyme-linked immunosorbent assay (ELISA)

Mice serum samples were diluted 1:100 with assay buffer for anti-dsDNA antibody and anti-cardiolipin antibody detection. Mouse Anti-dsDNA IgG ELISA kit and Mouse Anti-cardiolipin IgG ELISA Kit (#5210, #5515, respectively, Alpha Diagnostic Intl. Inc.) were used following the manufacturer’s protocols.

### 2.8 Statistical analysis

An *a priori* power analysis was conducted using G*Power to detect significant differences (*P* < 0.05, 1-ß = 80%) in responses to ACh 1) between MRL/lpr and MRL/MpJ strains; and 2) between lpr treatment and control groups. Based on preliminary data, N = 4 mice/strain are needed to detect a 30% difference between lpr and MpJ, and N = 13 mice/group are needed to detect a 15% difference between treatment and control groups. Concentration-response data were compared using two-way repeated measures ANOVA followed by Šídák’s multiple comparisons test. Differences in body weight, heart and spleen weights, spleen lengths, and protein expression were analyzed using Two-way ANOVA (strain × group) followed by Tukey posthoc test to examine group differences. Outliers were detected using the ROUT method (Q = 1%) and removed if identified. All statistics analyses were performed using Prism 10 (GraphPad Software, CA, United States), and JMP Pro 16 (SAS, Cary, NC, United States). Statistical significance was set at *p* < 0.05.

## 3 Results

### 3.1 Rapamycin reduces lupus phenotypes in MRL/lpr mice

MRL/lpr mice can develop SLE-like phenotypes such as autoantibodies to nuclear antigens and cardiolipin, nephritis, and splenomegaly. To assess if rapamycin treatment has preventive effects on developing disease phenotypes, both MRL/lpr and MRL/MpJ mice were treated with rapamycin starting at 9 weeks of age when they had no overt lupus manifestations developed (Table 1). After 8 weeks of rapamycin treatment, no significant difference was found in body weight across the groups (Table 1). There were significant differences in SLE-specific phenotypes. The spleen length was significantly greater in lpr_Control compared to MpJ_Control (*p* < 0.0001). Spleens from the mice treated with rapamycin (lpr_Rapamycin) were significantly shorter when compared to the lupus control group (*p* < 0.0001) (Table 1). Spleen weight-to-body weight (SW/BW) of lpr_Control mice was significantly heavier than MpJ_Control mice (*p* < 0.0001), which shows the development of splenomegaly in lupus mice. SW/BW from lpr_Rapamycin mice was significantly smaller compared to lpr_Control mice (*p* < 0.0001). The weight of the kidneys was significantly heavier in lpr_Control when compared to MpJ_Control (*p* < 0.0001). Rapamycin had a significant effect on reducing the kidney weights in lpr_Rapamycin compared to lpr_Control group (*p* < 0.0001). Anti-cardiolipin antibody levels of lpr_Control were significantly higher than all the other groups (*p* < 0.0001). Anti-dsDNA antibodies level from the serum of lpr Control was significantly higher than that of MpJ_Control (*p* = 0.0242). Also, lpr_Rapamycin group had reduced anti-dsDNA antibody levels compared to lpr_Control group.

**TABLE 1:** Body mass, tissue mass, and autoantibodies in untreated and treated lupus-prone and healthy control mice.

	lpr_Con	lpr_Rapa	MpJ_Con	MpJ_Rapa
Body weight (g)	38.2 ± 1.1	35.4 ± 0.8	37.9 ± 1.0	35.4 ± 0.7
HW/BW (mg/g)	4.53 ± 0.14	3.69 ± 0.39*	4.31 ± 0.11	4.14 ± 0.12
SW/BW (mg/g)	14.49 ± 0.97	5.58 ± 0.62*	2.33 ± 0.07*	2.29 ± 0.15*
Spleen length (cm)	2.9 ± 0.1	2.1 ± 0.1*	1.5 ± 0.0*	1.6 ± 0.0*
Kidney weight (mg)	263.4 ± 11.4	203.3 ± 4.4*	207.2 ± 4.6*	202.3 ± 5.5*
Anti-cardiolipin Ab (kU/mL)	2.32 ± 0.33	0.51 ± 0.13*	0.39 ± 0.06*	0.36 ± 0.05*
Anti-dsDNA Ab (kU/mL)	112.1 ± 11.9	73.6 ± 14.0	56.9 ± 16.2*	11.3 ± 4.3*

BW, body weight; HW, heart weight; SW, spleen weight; N = 9–12/group; data are means ± SEM; *, significantly different from lpr_Control (*p* < 0.05).

The association between molecular parameters (Anti-dsDNA antibodies and anti-cardiolipin antibodies) and SLE phenotypes is shown in Table 2. Both anti-cardiolipin and anti-dsDNA antibody levels were associated with parameters of splenomegaly. Spleen weight was positively correlated with both disease activities of lupus represented by anti-cardiolipin and anti-dsDNA antibodies (*p* < 0.0001). Kidney weight was positively linked with anti-cardiolipin antibodies and anti-dsDNA antibodies (*p* < 0.0001) which may suggest a potential relationship between biomarkers of SLE and lupus nephritis.

**TABLE 2:** Correlation (r) between SLE parameters and anti-cardiolipin, anti-dsDNA antibodies.

Variables	Anti-cardiolipin Abs r, (*p*-value)	Anti-dsDNA Abs r, (*p*-value)
SW/BW	0.78, (<0.0001)	0.75, (<0.0001)
Spleen length	0.76, (<0.0001)	0.76, (<0.0001)
Kidney weight	0.74, (<0.0001)	0.51, (0.011)
ACh max	−0.75, (<0.0001)	−0.52, (0.0092)

SW/BW, spleen weight to body weight.

### 3.2 SLE mice had increased mTOR activation compared to the healthy mice

**FIGURE 1:**
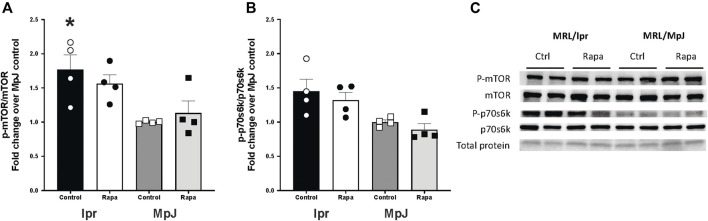
mTOR activation in liver of lupus mice. Expression and activation of mammalian target of rapamycin (mTOR) target **(A)**, and p-ribosomal s6 kinase **(B)** in livers from MRL/lpr and MRL/MpJ mice with and without rapamycin treatment. **(C)** shows Western blot images of phosphorylated mTOR, total mTOR, phosphorylated p70s6k, total p70s6k, and total protein from ponceau stain. Values are means ± SEM, n = 4–8 per group. *, Significantly different from MpJ_Control, *p* < 0.05.

The mTOR activation and a downstream marker of mTOR activity, S6 kinase from livers are shown in Figure 1. Phosphorylation of mTOR from the liver (Figures 1A, C) was significantly higher in lupus mice (lpr_Control, 1.76 ± 0.21 a.u.) compared to the healthy control group (MpJ_Control, 1.00 ± 0.01 a.u.) (*p* = 0.01). Although the mTOR activation from livers was not different after the 8 weeks of rapamycin treatment, these results imply that SLE results in enhanced activation of mTOR (Figure 1A). Phosphorylation of p70s6 kinase in lpr_Control (1.45 ± 0.17 a.u.) was greater than MpJ_control (1.00 ± 0.03 a.u., *p* = 0.07), which shows a consistent trend with mTOR activation (Figures 1B, C).

### 3.3 mTORC1 inhibition by rapamycin treatment prevented endothelial dysfunction

**FIGURE 2:**
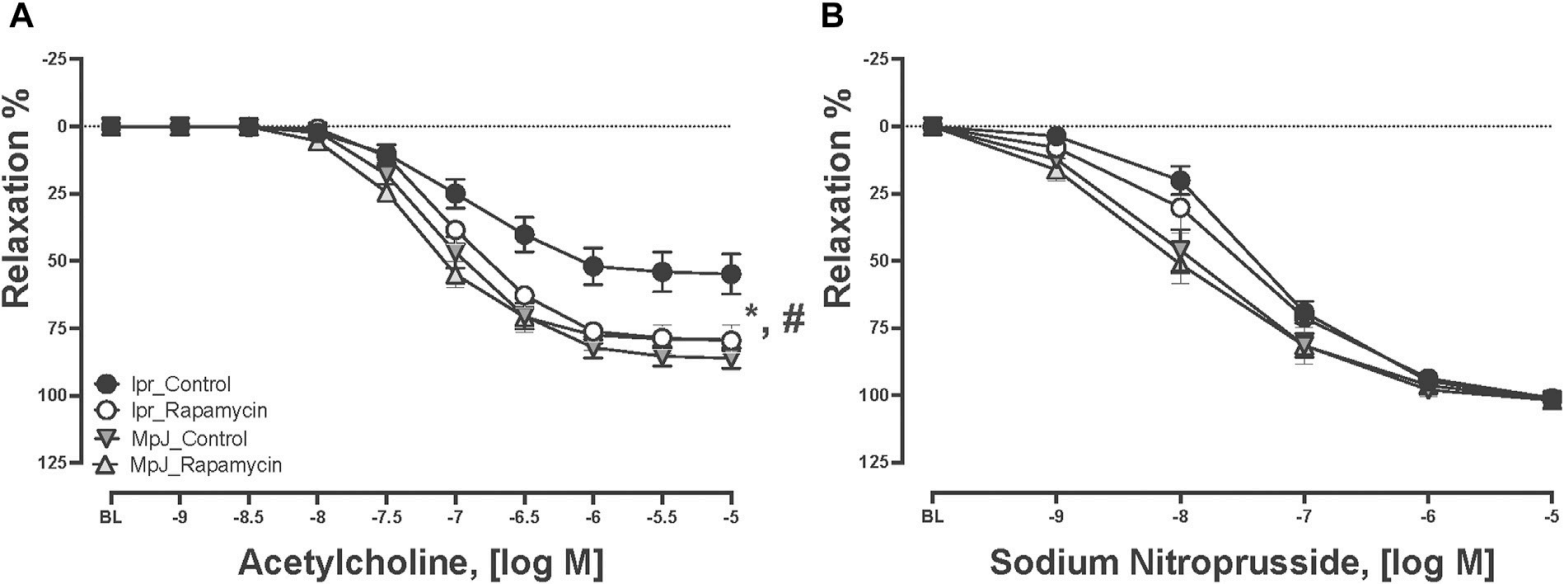
Rapamycin prevents endothelial dysfunction in mice. Relaxation responses to increasing concentration of ACh **(A)** and SNP **(B)** in thoracic aortas from MRL/lpr and MRL/MpJ mice with and without rapamycin treatment. Values are means ± SEM, n = 9–11 per group. BL, baseline after 70% maximal contraction with phenylephrine. *, Significantly different between lpr_Control and MpJ_Control, *p* < 0.05. ^#^, Significantly different between lpr_Control and lpr_Rapamycin, *p* < 0.05.

Mice with SLE can develop endothelial dysfunction which is a potential precursor of high risk of CVD. Concentration-response curves to ACh and SNP are in Figure 2. Thoracic aortas from each group were pre-contracted 70% of their maximal contraction. All vessels relaxed to ACh, however, vasorelaxation was significantly impaired in the lpr_Control group. Maximal vasorelaxation responses to ACh were significantly reduced in the lpr_Control (51.7% ± 6.7%, n = 11) compared to the MpJ_Control (86.7% ± 3.7%, n = 11, *p* < 0.0001). We assessed if rapamycin treatment could prevent endothelial dysfunction in lupus. Maximal vasorelaxation responses were significantly greater in the lpr_Rapamycin (79.9% ± 3.7%, n = 10) after 8 weeks of treatment when compared to the lpr_Control (*p* = 0.002). This result suggests that rapamycin helped prevent the lupus mice from developing endothelial dysfunction. Maximal vasorelaxation responses to SNP were not significantly different across any groups, suggesting that impaired responses to ACh in SLE mice were not due to the impaired vascular smooth muscle function.

### 3.4 Strain differences were found in vasocontractile responses to vasoconstrictors

**FIGURE 3:**
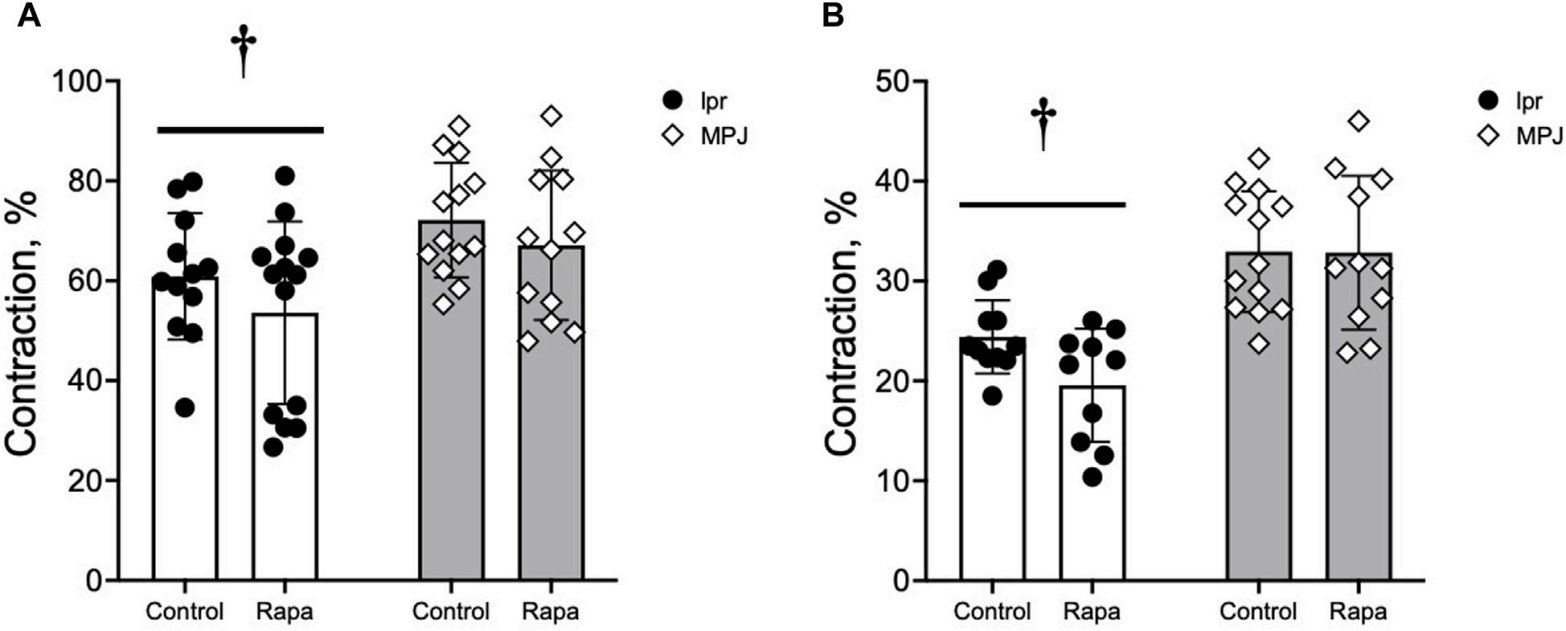
Vascular contraction is reduced in the aorta from lupus mice. Maximal contraction (%) calculated from the concentration-dependent contractile response to PE **(A)** and KCl **(B)**. Data represent mean ± SEM, n = 9–11 per group. ^†^, Significantly different from MpJ, *p* < 0.05.

The vasocontractile responses to PE and KCl were measured. Contractile responses to PE and KCl are shown in Figure 3. Maximal contractile responses to PE were significantly reduced in the aorta from lpr mice compared to MpJ mice, regardless of treatment (Figure 3A). Similarly, maximal responses to KCl were also significantly reduced in the aorta from lpr mice compared to MpJ mice (Figure 3B). As a marker of potency and efficacy, area under the curve (AUC) was determined for PE and KCl. Similar to maximal contractile responses, AUC was significantly lower in aorta from lpr mice compared to MpJ regardless of treatment (data not shown).

### 3.5 Anti-dsDNA and anti-cardiolipin antibodies are associated with impaired vasomotor function, endothelial dysfunction and SLE phenotypes

**FIGURE 4:**
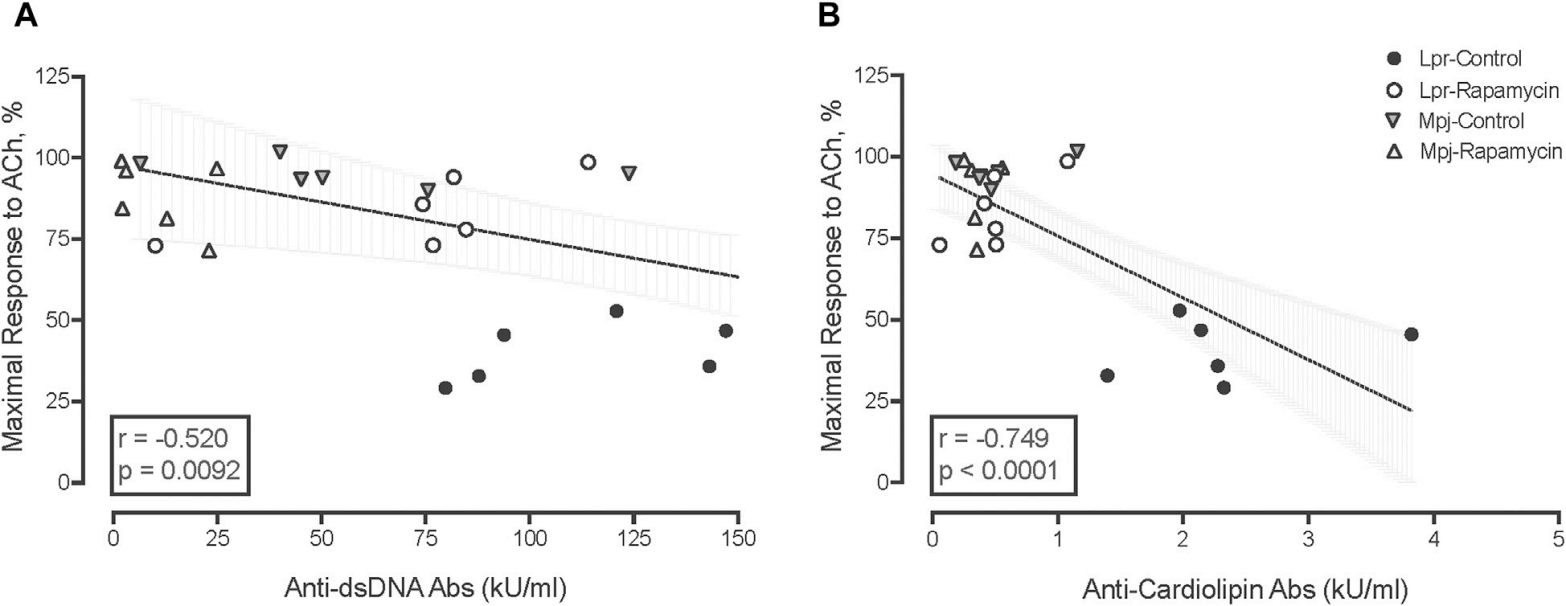
Negative correlation between anti-dsDNA Ab levels **(A)**, and anti-cardiolipin Ab levels (kU/mL) **(B)** with maximal response to ACh (%). The box contains the correlation coefficient (r) and *p* value.

Both anti-dsDNA antibodies and anti-cardiolipin antibodies were negatively correlated with maximal vasorelaxation responses to ACh (r: −0.520, *p* = 0.0092, Figure 4A and r: −0.749, *p* < 0.0001, Figure 4B). SLE phenotypes also were negatively correlated with maximal vasorelaxation response to ACh (correlation coefficient r ranging from −0.75 to −0.45 with *p*-value from <0.0001 to 0.002, data not shown) suggesting that the mice with higher disease activity have worse endothelial function. Anti-dsDNA antibodies were negatively correlated with maximal contractile responses and AUC of PE and KCl-mediated vasocontraction (r: −0.55 and −0.51, *p* = 0.0067 and 0.02). SLE phenotypes were negatively correlated with maximal contractile response to PE and KCl, (correlation coefficient r ranging from −0.55 to −0.33 with *p*-value from 0.0002 to 0.02, data not shown) suggesting that disease activity is associated with altered vasomotor function not just endothelial dysfunction. Body weight and heart weight to body weight were not significantly correlated with responses to vasoactive agents, suggesting that impaired vasomotor function in SLE is specific to lupus disease activity.

**FIGURE 5:**
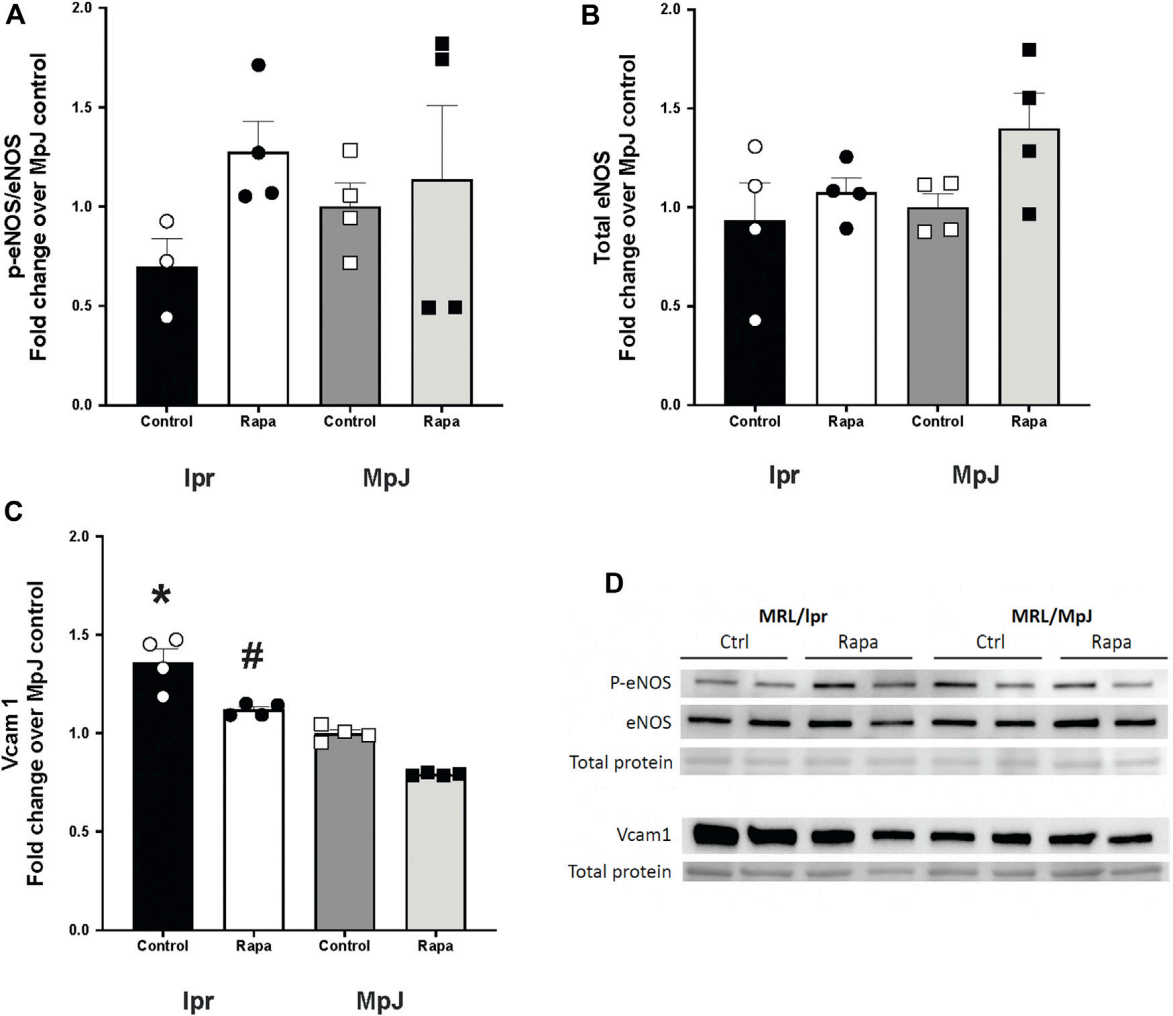
Effect of rapamycin on endothelial nitric oxide synthase (eNOS) and Vcam1. Densitometry analysis of the Western blot for p-eNOS/eNOS **(A)**, total eNOS **(B)**, and Vcam1 **(C)** in thoracic aortas from MRL/lpr and MRL/MpJ mice with and without rapamycin treatment. **(D)** images of Western blot of phosphorylated eNOS, total eNOS, Vcam1, and total protein from ponceau stain. Values are means ± SEM. N = 4 mice per group. *, Significantly different from MpJ_Control, *p* < 0.05. ^#^, Significantly different from lpr_Control, *p* < 0.05.

To determine the effect of rapamycin on endothelial nitric oxide synthase (eNOS) activation, which could improve endothelial function, we assessed the protein level of eNOS and its activation (Figures 5A, D). Phosphorylation of eNOS at Ser 1177 is consistent with eNOS activation. The phosphorylation of eNOS was lower in the lupus group (lpr_Control, 0.69 ± 0.14 a.u.) when compared to the healthy control group (MpJ_Control, 1.00 ± 011 a.u.). Although it was not statistically different, phospho-eNOS:total eNOS ratio increased 46% after the rapamycin treatment (lpr_Rapamycin, 1.27 ± 0.15 a.u.). Total eNOS was similar across the groups (Figures 5B, D). The protein level of the vascular inflammatory marker, vascular cell adhesion protein1 (Vcam1) also was measured in thoracic aortas (Figures 5C, D). Vcam1 level was significantly higher in the lpr_Control (1.36 ± 0.06 a.u.) when compared to MpJ_Control (1.00 ± 0.01 a.u.) (*p* = 0.001), suggesting the increased inflammatory responses in SLE in the blood vessel. Vcam1 from the lpr_Rapamycin (1.12 ± 0.01 a.u.) was significantly lower compared to lpr_Control (*p* = 0021).

### 3.6 Enhanced mTOR activation with SLE was prevented by rapamycin treatment

**FIGURE 6:**
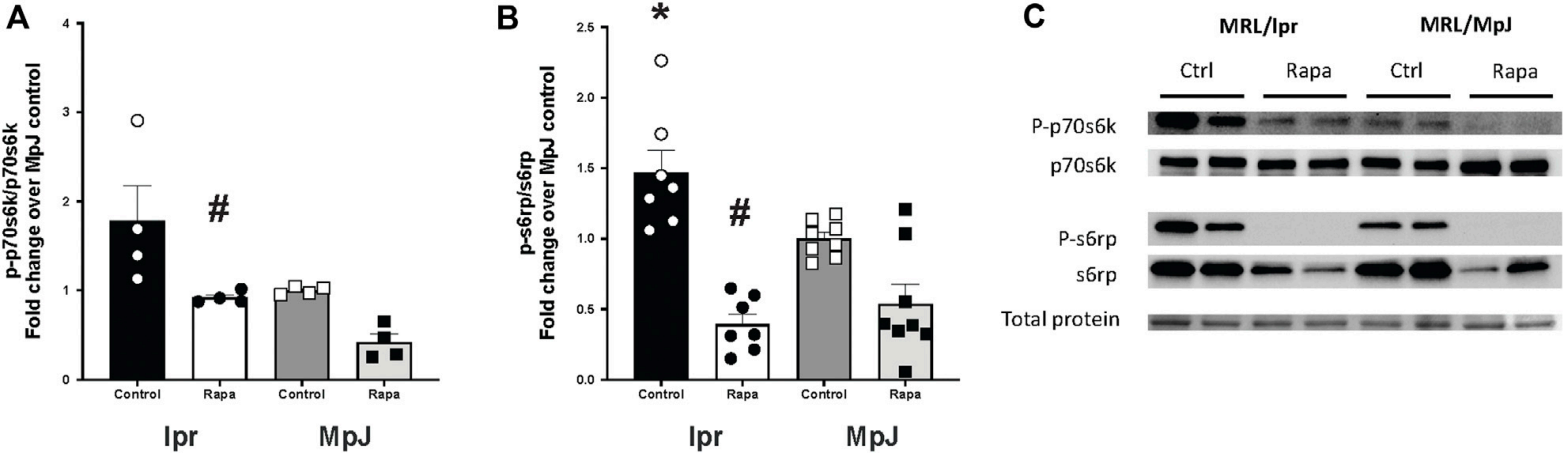
Enhanced mTOR activation with SLE was prevented by rapamycin treatment. Densitometry analysis of the Western blot for p-p70s6k/p70s6k **(A)**, and p-s6rp/s6rp **(B)** in thoracic aortas from MRL/lpr and MRL/MpJ mice with and without rapamycin treatment. **(C)** images of Western blot of phosphorylated p70s6k, total p70s6k, phosphorylated s6rp, total s6rp, and total protein from the ponceau stain. Values are means ± SEM. N = 4–8 mice per group. *, Significantly different from MpJ_Control, *p* < 0.05. ^#^, Significantly different from lpr_Control, *p* < 0.05.

S6 protein kinase (P70s6k) was highly activated in lpr_Control (1.78 ± 0.39 a.u.) compared to MpJ_Control (1.00 ± 0.02 a.u.) (Figures 6A, C). With rapamycin treatment, there was a significant reduction in the phosphorylation of p70s6k in the lpr_Rapamycin (0.92 ± 0.03 a.u.) group compared to lpr_Control (1.78 ± 0.39 a.u.) (*p* = 0.04). S6 ribosomal protein (S6rp), another downstream marker of mTORC1 was significantly increased in lpr_Control (1.46 ± 0.15 a.u.) compared to MpJ_Control (1.00 ± 0.04 a.u.) (*p* < 0.0001) (Figures 6B, C). The phosphorylation of s6rp was significantly reduced in lpr_Rapamycin (0.39 ± 0.07 a.u., *p* < 0.0001), which confirmed the effect of rapamycin on inhibiting the mTORC1 activity (Figures 6B, C). These results suggest that mTORC1 is highly activated in the aorta of SLE mice and is inhibited by rapamycin.

### 3.7 The effect of mTOR inhibition in lupus mice by rapamycin treatment on mitophagy markers

**FIGURE 7:**
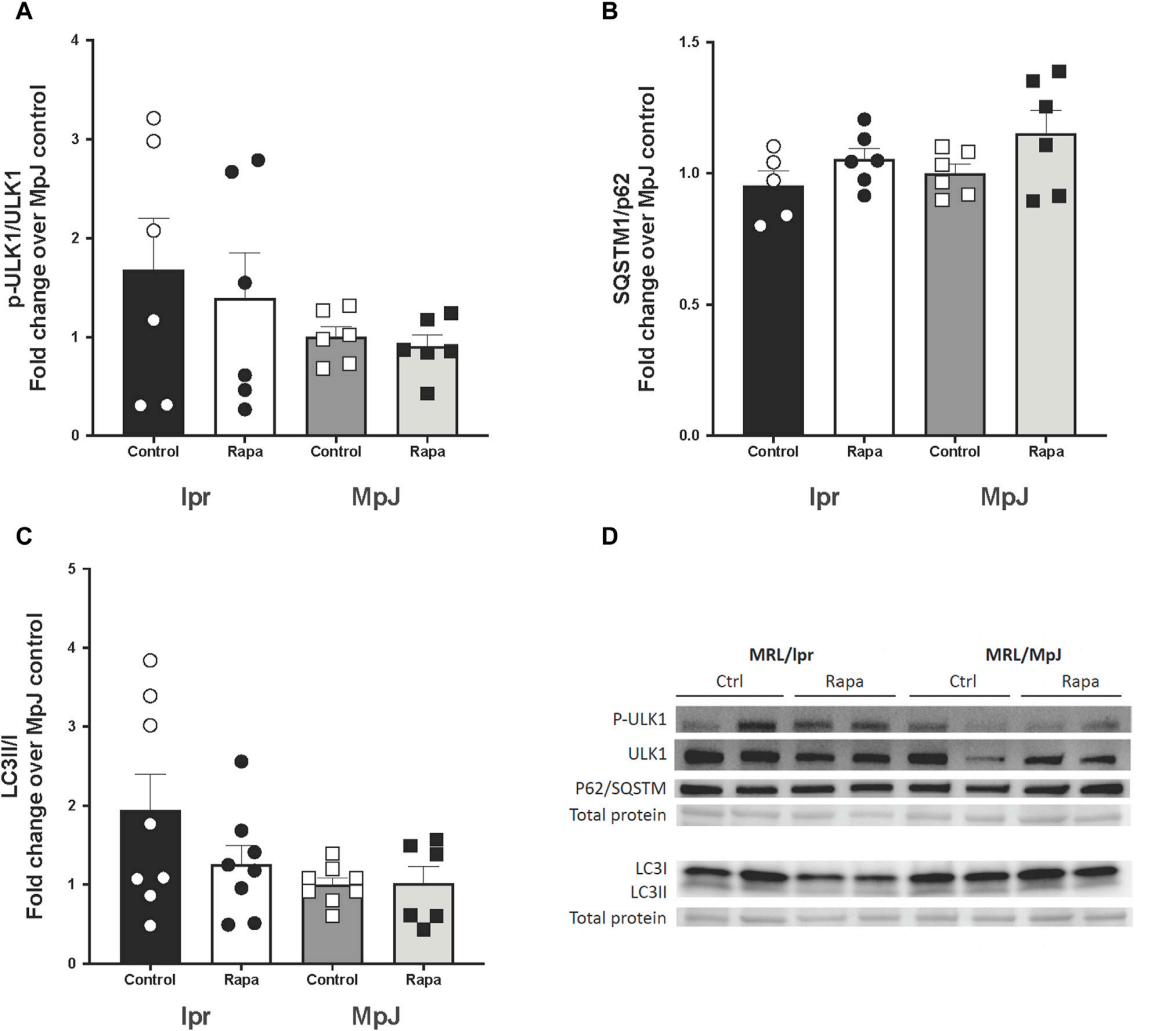
The effect of mTOR inhibition in lupus mice by rapamycin treatment on mitophagy markers. Densitometry analysis of the Western blot for p-ULK1/ULK1 **(A)**, SQSTM1/p62 **(B)**, and LC3 II/I **(C)** in thoracic aortas from MRL/lpr and MRL/MpJ mice with and without rapamycin treatment. **(D)** shows the Western blot images. Values are means ± SEM. N = 5–8 mice per group.

Inhibition of mTOR activation can enhance mitophagy. SQSTM1/p62, LC3II/I, and p-ULK1/ULK1 were measured as markers of mitophagy (Figure 7). High mTOR activity prevents ULK1 activation by phosphorylating ULK ser757 which inhibits autophagy. lpr_Control (1.42 ± 0.66 a.u.) had higher phosphorylation of ULK1 when compared with MpJ_Control (0.72 ± 0.28 a.u.) which may suggest a reduced influx of autophagy in lupus mice. lpr_Rapamycin (1.00 ± 0.13 a.u.) group had reduced phosphorylation of ULK1 when compared with lpr_Control (Figures 7A, D), suggesting the effect of rapamycin increasing the influx of autophagy by attenuating phosphorylation of ULK1. There were no significant differences in the protein expression of p62 across any groups (Figures 7B, D). Protein expression of LC3II/I was nearly 2-fold higher in lpr_Control (1.94 ± 0.46 a.u.) when compared to MpJ_Control (1.00 ± 0.09 a.u.). LC3II/I ratio was lower in lpr_Rapamycin (1.26 ± 0.24 a.u.) than lpr_Control, which suggests that rapamycin might have increased the influx of mitophagy, thus reducing LC3II remaining in the aorta (Figures 7C, D).

## 4 Discussion

mTOR activation regulates mitochondrial homeostasis by stimulating mitochondrial biogenesis, and dynamics, and by inhibiting mitophagy. Higher mTORC1 activity has been reported in SLE patients and lupus-prone mice. Our results from female lupus-prone mice (MRL/lpr) showed higher activation of mTOR in the liver and thoracic aorta. Endothelial function also was impaired in lpr mice compared to healthy control mice (MRL/MpJ). Inhibiting mTOR with rapamycin prevented endothelial dysfunction, attenuated lupus disease phenotypes and altered mitophagy markers in lupus mice. The results of the present study provide evidence of the beneficial effect of rapamycin on preventing endothelial dysfunction in SLE.

### 4.1 Rapamycin and endothelial function

**FIGURE 8:**
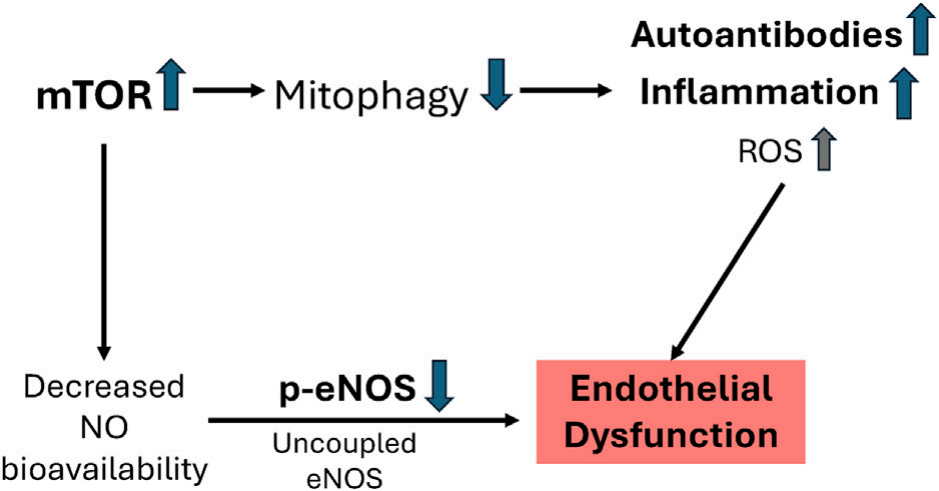
Proposed mechanism for endothelial dysfunction in MRL/lpr mice. Increased mTOR activity (Figure 6) in lupus inhibits mitophagy leading to increased autoantibodies (Figure 4), inflammatory responses (Figure 5), and oxidative stress. Increased mTOR decreases NO bioavailability (e.g., decreased p-eNOS/eNOS) (Figure 5) leading to impaired vascular function (Figure 2).

Work by Perl and colleagues (Fernandez et al., 2009; Caza et al., 2014; Oaks et al., 2016) demonstrated that mTOR activity is increased in SLE and contributes to disease activity. Their work showed that increased oxidative stress and nitric oxide induce mitochondrial hyperpolarization which is a signal to increase mTOR activity (Nagy et al., 2003; Nagy et al., 2004; Fernandez et al., 2009). Increased mTOR inhibits mitophagy, contributing to an accumulation of mitochondria which predisposes to cellular necrosis and release of inflammatory mediators (Caza et al., 2014; Oaks et al., 2016). In aging, increased mTOR activity is associated with increased oxidative stress, reduced nitric oxide bioavailability, and impaired endothelial function (Rajapakse et al., 2011; Yepuri et al., 2012; Lesniewski et al., 2017; Islam et al., 2024). Therefore, we hypothesized that endothelial function in lupus mice would be impaired due to mTOR-associated decreases in nitric oxide signaling and reduced mitophagy (Figure 8). In the current study, lpr control mice had significantly impaired endothelial function compared to MpJ control mice. Our finding shows that the maximal vasorelaxation in lpr_Control mice was 50% lower than that of MpJ_Control. This finding is consistent with previous results of endothelial dysfunction in lupus mice (Bagi et al., 2006; Knight et al., 2015; Furumoto et al., 2017; Robles-Vera et al., 2020). For example, MRL/lpr mice exhibited an endothelial dysfunction ranging from 60% to 75% of endothelium-dependent vasorelaxation in the thoracic aorta (Marczynski et al., 2021; Shi et al., 2023). Also, thirty-six-week-old NZBWF1 mice reported impaired endothelial function in both carotid arteries and thoracic aortas (Ryan and McLemore, 2007; Thacker et al., 2010). However, the mechanism of endothelial dysfunction is unclear.

In T-cells of lupus patients and mice, elevated mTOR activity contributes to the pathogenesis of disease (Perl, 2010; Caza et al., 2014; Oaks et al., 2016). mTORC1 has also been shown to regulate endothelial function (Rajapakse et al., 2011; Yepuri et al., 2012; Reho et al., 2019; Islam et al., 2024). To determine the role of mTOR signaling in endothelial function in SLE we inhibited mTOR with rapamycin. Thoracic aortas from lupus mice treated with rapamycin showed greater maximal vasorelaxation responses to acetylcholine, supporting the beneficial effect of inhibiting mTORC1 with rapamycin on endothelial function in SLE. We also found that the downstream markers of mTORC1 were significantly higher in the lupus mice than in the healthy control mice. The levels of these markers decreased with rapamycin treatment, demonstrating the effectiveness of rapamycin, an inhibitor of mTOR signaling. Collectively, these results suggest that elevated mTOR signaling contributes to endothelial dysfunction in SLE which can be prevented by treating with rapamycin.

One proposed mechanism for endothelial dysfunction with chronically elevated mTOR is a disruption of endothelial nitric oxide signaling. Increased S6K activity in endothelial cells leads to oxidative stress and decreased nitric oxide bioavailability (e.g., uncoupled eNOS), with variable effects on eNOS protein level (Rajapakse et al., 2011; Yepuri et al., 2012; Islam et al., 2024). Deletion of mTOR in endothelial cells prevents these deleterious effects (Islam et al., 2024). These effects can also be attenuated or reversed by rapamycin (Rajapakse et al., 2011). To assess the effects of SLE and rapamycin on NO signaling we measured the protein level of eNOS and assessed eNOS activity by examining the ratio of phosphorylated eNOS to total eNOS. Phospho-eNOS:total eNOS ratio was not significantly different between lpr_Control and MpJ_Control, although it was higher in MpJ_Control. The rapamycin-treated lpr group had higher phosphorylated eNOS compared to the untreated lpr group as well, suggesting that eNOS activity was altered in aorta from lupus mice and with rapamycin treatment. Although the current study and others support the beneficial effects of rapamycin on endothelial function, others showed a negative effect of rapamycin on endothelial function causing a reduction of phosphorylated eNOS (Jeanmart et al., 2002; Reineke et al., 2015; Wang et al., 2022). In addition, increased NO in T-cells from lupus patients and mice contributes to the activation of mTOR and accumulation of mitochondria, promoting SLE disease activity (Nagy et al., 2003; Nagy et al., 2004; Fernandez et al., 2009). Those results suggest that elevated NO contributes to lupus disease activity. mTOR activity is also elevated in renal endothelial cells of lupus patients and mice (Mao et al., 2022; Huang et al., 2024) and could serve as a biomarker and predictor of disease progress in lupus nephritis (Mao et al., 2022). However, maintaining NO levels in renal endothelial cells is beneficial for reducing the symptoms of lupus nephritis (Gilkeson et al., 2013). Thus, the interaction between elevated mTOR and nitric oxide signaling in lupus might be cell-type specific. In the current study, inhibition of mTOR with rapamycin improved endothelial function in SLE mice. Further studies supporting the evidence of rapamycin altering NO bioavailability are necessary to determine the underlying mechanism of rapamycin on this pathway. Interestingly, in the liver mTOR is associated with the generation and release of autophospholipid antibodies, including anti-ß_2_-glyocprotein I antibodies (Oaks et al., 2016). Anti-ß_2_-glyocprotein I antibody signaling in endothelial cells has been shown to inhibit autophagy by activating mTOR and NO pathways (Zhang et al., 2021). Furthermore, anti-ß_2_-glyocprotein I binding to LRP8 in lipid rafts can decrease phospho-eNOS and NO, suggesting that LRP8 signaling could be a potential mechanism for impaired endothelial function in lupus as well as antiphospholipid syndrome (APS) (Riitano et al., 2023). Future research should investigate the contribution of LRP8 signaling to endothelial dysfunction in SLE.

Rapamycin functions as a potent anti-inflammatory drug via multiple mechanisms. It has been widely applied in clinical populations with inflammation-related diseases (Madonna et al., 2015). Rapamycin has been used in SLE to ameliorate inflammation in liver, kidney, spleen, skin, etc (Warner et al., 1994). Our study extends the results of previous literature to assess the anti-inflammatory effect of rapamycin on the blood vessels of SLE mice. Studies have shown that Vcam1 levels are elevated in serum, urine and kidneys of SLE patients (Santos et al., 2012; Davies et al., 2021) and patients with active lupus nephritis (Svenungsson et al., 2008). Elevated Vcam1 is indicative of an inflammatory response within the vasculature. Although it is associated with endothelial dysfunction (Hwang et al., 1997), protein level of Vcam1 is underexplored in the vasculature in SLE models. Our study measured protein Vcam1 from the aorta of lupus and healthy mice to expand the results from the previous literature that determined the increased level of Vcam1 in heart, brain, kidney, and lung of SLE mice (McHale et al., 1999). Lupus mice had higher protein levels of Vcam1 in aorta compared with MpJ healthy control mice, suggesting the high inflammatory responses in the vessels of lupus mice. The rapamycin-treated mice had significantly lower Vcam1 expressed in vessels compared to untreated lupus mice. Our findings imply that rapamycin prevented the endothelial dysfunction of SLE by attenuating inflammation in the blood vessels.

In lupus T-cells, mitochondrial dysfunction including mitochondrial hyperpolarization, mitochondrial accumulation, and impaired mitophagy is mediated by increased Rab4A and its interaction with mTOR (Fernandez et al., 2009; Caza et al., 2014; Oaks et al., 2016; Huang et al., 2024). In multiple cells, those changes are associated with increased mTOR activity and elevated levels of anti-cardiolipin and anti-ß_2_-glyocprotein I autoantibodies and are inhibited by rapamycin (Oaks et al., 2016; Huang et al., 2024). In blood vessels from hypertensive rats and diabetic mice, impaired autophagy/mitophagy, characterized by increased SQSTM/p62 (McCarthy et al., 2019; Zhao et al., 2022) and LC3 II (Zhao et al., 2022), was associated with impaired endothelial function. Restoring autophagy/mitophagy improved vascular relaxation in mesenteric arteries and aorta (McCarthy et al., 2019; Zhao et al., 2022). Although the efficacy of rapamycin for attenuating disease activity in the clinical population has been reported in organ/tissue/cells like the kidney, T cells, and liver (Kato and Perl, 2014; Liu et al., 2015; Lai et al., 2018), the effect of modulating mitophagy/autophagy with rapamycin in blood vessels from lupus patients or mice is unclear. In the current study protein level of markers for mitophagy/autophagy were measured in the thoracic aorta of SLE mice to determine the effect of rapamycin on that signaling pathway relative to endothelial function.

ULK1, a critical regulator of clearance of mitochondria (Kundu et al., 2008), is regulated by mTOR (Dossou and Basu, 2019), which inhibits ULK1 phosphorylation (Nazio et al., 2013). Our results indicate that inhibiting mTORC1 decreased ULK1 phosphorylation in the thoracic aorta from the rapamycin-treated lpr mice. Reduced phosphorylation of ULK1 might suggest activated autophagosome causing better clearance of damaged cells in rapamycin-treated lupus mice. The attachment of mitochondria to the autophagosome membrane via p62/SQSTM and LC3II play a role in the process of mitophagy (Jin and Youle, 2012). Changes in p62/SQSTM and LC3II/I have been associated with endothelial cell autophagy/mitophagy and endothelial function in mesenteric arteries and aorta from rats and mice, respectively (McCarthy et al., 2019; Zhao et al., 2022). Therefore, the protein level of LC3II/LC3I, one of the markers of mitophagy, was also measured in the thoracic aorta from lupus-prone mice. We found an increased level of LC3II/LC3I ratio from the lupus control mice compared to MpJ control mice. Deficient mitophagy causes the accumulation of ubiquitinated proteins and LC3 on the surface of mitochondria (Kageyama et al., 2014; Yamada et al., 2019), which would explain the defective mitophagy in our lpr control mice with elevated LC3II/LC3I. The elevated level of LC3II to LC3I ratio indicates the accumulation of autophagosomes, but it does not guarantee autophagic degradation and is hindered from processed through a lysosomal phase (Youle and Narendra, 2011). LC3II/LC3I was reduced in rapamycin-treated lupus mice, suggesting that autophagosomes have fused with lysosomes and thus degraded their contents. SQSTM/p62 level were the same across the groups. A similar result was reported previously for the mouse embryonic fibroblast cells treated with rapamycin (at 1 μM and 10 nM). No changes in p62 were reported after 2, 8, and 24 h. Therefore, changes in LC3II/I can occur independent of changes in protein levels of p62 (de Wet et al., 2021).

### 4.2 Rapamycin and lupus phenotypes

SLE is an autoimmune disease characterized by the production of antibodies to various antigens (D’Andrea et al., 1996; Delgado Alves et al., 2003; Lartigue et al., 2005). An increasing number of mitochondria-derived damage-associated molecular pattern (mtDAMPs) have been linked to SLE (Xu et al., 2020; Koenig and Buskiewicz-Koenig, 2022). Dysfunctional mitophagy can cause a release of mtDAMPs, which causes inflammation to promote chronic vascular diseases such as atherosclerosis (Khwaja et al., 2021; Salnikova et al., 2021). We assessed the level of anti-cardiolipin antibodies and anti-dsDNA antibodies from serum of treated and untreated MRL/lpr and MRL/MpJ mice. Both autoantibodies were higher in lpr mice compared to MPJ and reduced significantly with rapamycin treatment compared to untreated lupus mice. Our results show both autoantibodies were negatively correlated with maximal endothelium-dependent vasorelaxation. This novel finding in mice supports a previous finding by Patino-Trives et al. in SLE patients (Patino-Trives et al., 2021). They showed that anti-dsDNA antibody titers and endothelial dysfunction parameters (hyperemia area and peak flow minus rest flow) were negatively correlated in lupus patients. Targeting these autoantibodies has been suggested as a possible treatment for lupus because these autoantibodies are associated with accelerated atherosclerosis (Marai et al., 2008; Patino-Trives et al., 2021). Other markers of disease activity (e.g., splenomegaly) were also negatively correlated with maximal endothelium-dependent vasorelaxation. Collectively, these markers were improved with rapamycin treatment. The negative correlations between lupus phenotypes and maximal ACh responses suggest that endothelial dysfunction in SLE is associated with disease activity. Furthermore, these results imply that the effect of rapamycin on reducing the lupus disease activity contributed to improved endothelial function of SLE.

### 4.3 Limitation and conclusion

Although we demonstrated that rapamycin improves endothelial dysfunction, we could not demonstrate a definitive mechanism. We hypothesized that rapamycin would increase mitophagy. However, the true flux of mitophagy in the current study is unclear. The true amount of LC3II between samples with and without the lysosome inhibitors can represent the level of mitophagy flux. In the previous literature, studies measured LC3II or LC3II/LC3I from the tissue with and without the lysosome inhibitors to block the autophagosome turnover to assess legitimate influx of autophagy/mitophagy (Youle and Narendra, 2011). Further studies can be done by measuring mitophagy or autophagy markers at different time points. One study showed distinct and dynamic changes in mitophagy markers, LC3II, and p62, within an acute duration of the intervention from 2 to 24 h (de Wet et al., 2021). Rapamycin intervention changed these markers in a time-dependent manner, causing a significant increase within 2 h and reducing the protein level down to baseline after 24 h. Non-significant changes in mitophagy markers shown in the current study may be because rapamycin treatment does not chronically change levels of mitophagy-associated proteins.

Rapamycin plays a pivotal role in clinical research targeting the attenuation of inflammatory responses. Patients receiving sirolimus for 3 months experienced improved disease symptoms with few serious side effects (Piranavan and Perl, 2021). More prolonged treatment with sirolimus also proved safe and effective for reducing disease activity in lupus patients (Lai et al., 2018). Sirolimus is also effective as an add-on treatment for decreasing clinical symptoms of SLE, suggesting that sirolimus can be part of a multi-drug treatment plan (Ding et al., 2024). Although this study contains some limitations, it addresses the gaps in previous studies of SLE, focusing on the effect of mTORC1 inhibition on vascular function, inflammation, and mitophagy in the blood vessels of SLE mice. Our finding showed a beneficial effect of rapamycin on improving endothelial function. Thus, the use of rapamycin alone or with immunosuppressant drugs may provide a potential therapeutic approach for reducing the disease activity and improving vascular function at the same time. Future clinical trials using rapamycin analogs should consider including measurements of endothelial function to translate the findings from the current study to patients with SLE.

## Data Availability

The original contributions presented in the study are included in the article/Supplementary Material, further inquiries can be directed to the corresponding author.
