# Supplementary material for: Beneficial effects of rapamycin on endothelial function in systemic lupus erythematosus
Source: Front Physiol. 2024 Aug 21;15:1446836. doi: 10.3389/fphys.2024.1446836 (PMC11372898; doi:10.3389/fphys.2024.1446836)
Supplement: Supplementary file 1 [file DataSheet1.pdf]

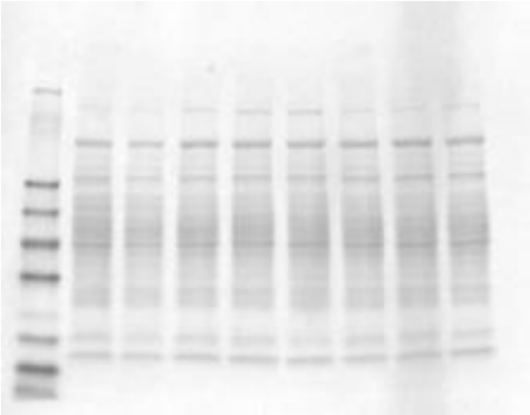

Supplemental Image 1. Total protein for liver (Figure 1)

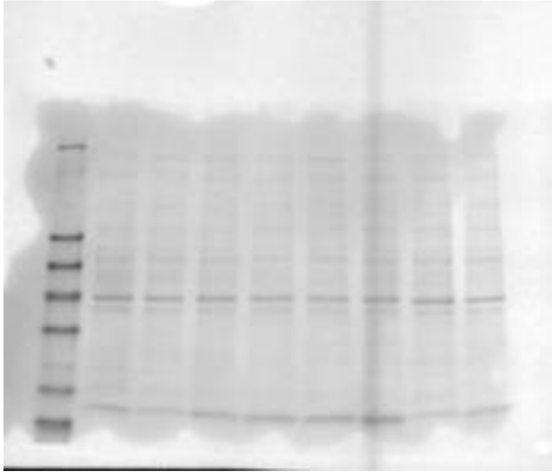

Supplemental Image 2. Total protein for p-eNOS/eNOS (Figure 5), p-s6rp/s6rp and p-p70s6k/p70s6k (Figure 6)

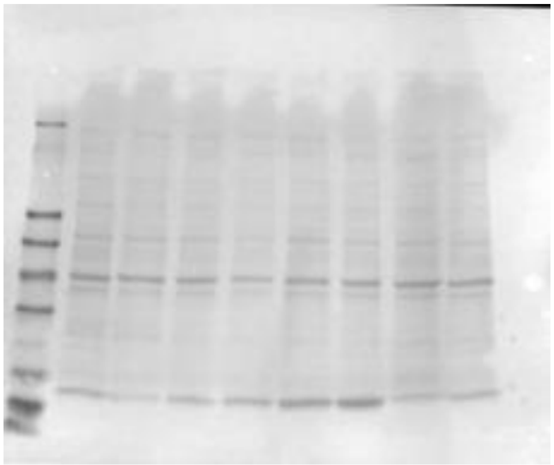

Supplemental Image 3. Total protein for Vcam1 (Figure 5), p-ULK/ ULK1, p62/SQSTM (Figure 7)

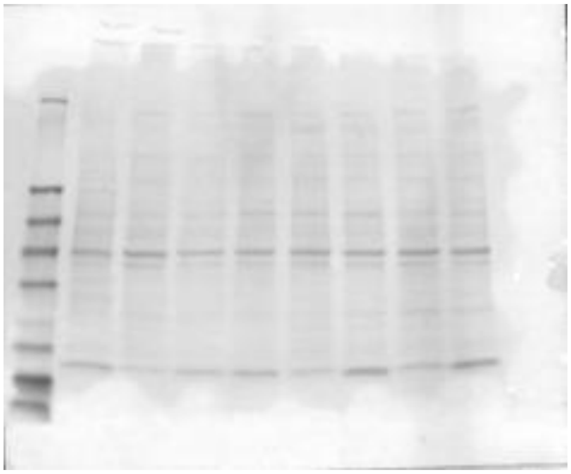

Supplemental Image 4. Total protein for LC3II/I (Figure 7)
